# Studying daily fluctuations of emotional effort among nurses of intensive care units: the establishment of latent profiles and its relationship with daily secondary traumatic stress and vitality

**DOI:** 10.3389/fpsyg.2024.1340740

**Published:** 2024-03-15

**Authors:** Jennifer E. Moreno-Jiménez, Miriam Romero, Luis Manuel Blanco-Donoso, Mercedes Hernández-Hurtado, Eva Garrosa

**Affiliations:** ^1^Faculty of Education and Psychology, Universidad Francisco de Vitoria, Madrid, Spain; ^2^Department of Biological and Health Sciences, European University of Madrid, Madrid, Spain; ^3^Faculty of Psychology, Autonomous University of Madrid, Madrid, Spain

**Keywords:** diary study, emotional effort, secondary traumatic stress, vitality, latent profile

## Abstract

**Introduction:**

Nursing professionals working in Intensive Care Units (ICU) face significant challenges that can result in secondary traumatic stress (STS). These challenges stem from witnessing patients’ suffering and managing difficult tasks (i.e. communication with patients’ relatives). Furthermore, these professionals encounter emotional demands, such as emotional effort, which is the dissonance between the emotion felt and the emotion that should be expressed to meet work expectations. Consequently, we aimed to investigate whether different profiles exist concerning nurses’ levels of emotional effort over a five-day period and whether these profiles are related to daily STS and vitality.

**Methods:**

The sample comprised 44 nursing professionals from ICUs in Spanish hospitals. They were assessed daily, using a package of questionnaires twice per day for five working days: a) immediately after their shift and b) at a later time after working.

**Results:**

The findings revealed three distinct profiles based on emotional effort levels: high (Profile 1), moderate (Profile 2), and low (Profile 3). These profiles were found to be negative predictors for both daily shattered assumptions and symptomatology.

**Discussion:**

This study underscores the importance of assessing daily emotional demands in an ICU setting. Such assessments are crucial for establishing preventive measures to help nursing professionals manage lower-level emotional demands.

## Introduction

Nursing professionals working in Intensive Care Units (ICU) are known to face various psychosocial risks (Nikbakht Nasrabadi et al., 2022). Working in an ICU entails working under pressure and tough stressors, which may lead to an increase in emotional exhaustion among healthcare professionals (Ballester-Arnal et al., 2016). Additionally, this could result in negative consequences related to trauma in the medium and long-term (Ariapooran et al., 2022). Specifically, the prevalence of secondary traumatic stress (STS) among nurses ranges from 7 to 67.64%, depending on the country and specific population studied (Partlak Günüşen et al., 2019). In Spain, studies conducted after the COVID-19 outbreak revealed alarming rates of STS, with 38% of nurses experiencing moderate or severe symptoms, consequently affecting their mental health (Martin-Rodriguez et al., 2022). Although no specific studies have updated the prevalence of STS among nurses, Luceño-Moreno et al. (2020) sampled 1,442 healthcare professionals and found that 56.6% suffered from posttraumatic stress. The prolonged strain and work stress experienced by these professionals may increase intention to quit their jobs, with a worldwide turnover rate of 27% (Xu et al., 2023). Thus, studying the occurrence of STS in nurses is undeniably relevant to provide a comprehensive understanding of their mental health and well-being.

Nurses are constantly in direct contact with patients, families, and other healthcare professionals, requiring effective communication skills (Foglia et al., 2010). Furthermore, the pandemic outbreak has increased patient load (Nikbakht Nasrabadi et al., 2022), increasing the scarcity of job resources and imposing several limitations on overcoming these difficulties (Blanco-Donoso et al., 2021; Moreno-Jiménez et al., 2021). This situation is further exacerbated by a decrease in the number of skilled nurses available to meet the growing demand (Nikbakht Nasrabadi et al., 2022). Similarly, Foglia et al. (2010) highlighted the need for nurses to simultaneously deal with technological and human skills. They are not only responsible for providing medical care, but also for communicating effectively and providing emotional support. These factors impose a difficult situation linked to the shortage of nursing professionals (Tolksdorf et al., 2022). These demands increase nurses’ emotional effort and are strongly related to psychosocial risks (Delgado et al., 2017).

### Emotional effort in nurses working in ICU

The emotional effort of nursing constitutes an emotional demand resulting from managing communication and relationships with families, patients, and colleagues (Delgado et al., 2017). Thus, nursing involves not only highly qualified skills related to caring for patients by using technology and appropriate knowledge, but also emotional skills to identify and regulate others’ emotions (Alonazi, 2020). Based on the work of Delgado et al. (2017), they discussed emotional labor to refer to this emotional demand in the work setting. Delgado et al. (2017) distinguished two types of emotional regulation: (a) deep acting, as a way of identifying and properly regulating one’s emotions, and (b) surface acting, because the emotions that need to be displayed to meet workplace rules do not match the emotions felt. The latter has the same meaning and characteristics as emotional effort (Quiñones-García et al., 2013), and has been strongly related to emotional exhaustion, burnout, and negative health consequences (Schmidt and Diestel, 2014). Hereafter, we refer to emotional effort to highlight the dissonance between meeting the rules of emotional expression and the emotions felt, as stated by surface acting.

As mentioned above, the direct relationship between emotional effort and exhaustion has been previously studied (Martínez-Iñigo et al., 2007). Prolonged emotional demands that require work in an ICU are strongly related to work-related stress (Zaghini et al., 2020), which could lead to emotional exhaustion (Bakker and Demerouti, 2017). However, few studies have focused on vitality as the opposite of emotional exhaustion (Bakker et al., 2023). Studies have shown that emotional demands can lead to positive outcomes when professionals possess proper emotion regulation strategies as emotional demands are more challenging than hindrances (Donoso et al., 2015). These facts could lead to feeling more vigorous at work, instead of being exhausted. Thus, we considered it highly interesting to test this hypothesis in an ICU.

The ICU context is also associated with the likelihood of developing other psychosocial risks such as STS (Van Mol et al., 2015; Barleycorn, 2019) due to the traumatic stimuli that nursing professionals must deal with. Shoji et al. (2015) found that burnout comes first as prolonged exposure to job-related stressors, whereas in the medium term, the likelihood of the appearance of STS in specific contexts is high as a result of specific stressors, such as the cycle of death and suffering, the contagion of patients and relatives, life-death decisions, and time pressure for attending patients. Thus, burnout and STS could be two different negative outcomes that are likely to appear in emotionally demanding contexts; however, few studies have focused on how STS develops (Moreno-Jiménez et al., 2022). To explain how this specific work context could lead to STS, we based our work on the conservation of resources theory (CoR; Hobfoll, 2001).

According to the CoR theory, demanding work settings can result in energy loss, similar to the concept of emotional exhaustion proposed by the Job Demands-Resources Model (Bakker et al., 2004). The emotional demands faced by nursing professionals can contribute to energy loss by depleting the resources required to cope with these demands (Le Blanc et al., 2001). This energy loss can occur across various work environments where an imbalance exists between demands and available resources (Demerouti et al., 2001). However, when this general energy loss occurs due to the depletion of a broad range of resources, it increases the likelihood of experiencing specific negative consequences owing to prolonged exposure to particular demands (Hobfoll, 2011). In the context of this study, the specific demands related to ICU may lead to STS (Van Mol et al., 2015).

### Emotional effort and secondary traumatic stress in nurses

Secondary traumatic stress refers to the natural response experienced by professionals through helping and caring for patients or victims of traumatic situations (Figley, 1999). Thus, advances in trauma research reveal that professionals in ICU are constantly exposed to traumatic stimuli, such as the cycle of death and suffering, communicating bad news to families, and high-pressure decision-making, among other stressors (Foglia et al., 2010). These stressors profoundly impact on the mental health of healthcare professionals. The COVID-19 outbreak has further intensified the traumatic stressors that could occur in an ICU, such as fear of contagion and close contact with death (Lázaro-Pérez et al., 2020). Recent studies have revealed that the prevalence of STS among ICU professionals is as high as 62% in some countries such as Iran (Ariapooran et al., 2022).

Advancements in studies on STS have revealed the importance of distinguishing among the three dimensions of this syndrome (Meda et al., 2011; Moreno-Jiménez et al., 2020), moving beyond the classical model proposed by Figley (1999). These dimensions provide a deeper understanding of the three levels of affection that could occur when referring to a trauma, as the traumatic stressors do not affect every professional in the same way, and can vary depending on individual and job resources (Donoso et al., 2015; Van Mol et al., 2015). The three dimensions are emotional, cognitive, and symptomatology.

The emotional dimension refers to compassion fatigue, which is similar to burnout among nurses (Steinheiser, 2018). Compassion fatigue appears as the stress of caring for others (Jakimowicz et al., 2018) and the depletion of empathetic skills as a result of chronic exposure to others’ suffering and a lack of self-care measures (Peters, 2018). Peters (2018) supported the idea that emotional exhaustion is a consequence of compassion fatigue. Thus, we hypothesized that the emotional demand for emotional effort could have a direct and positive relationship with the development of compassion fatigue, similar to emotional exhaustion.

The cognitive dimension, known as shattered assumptions, refers to the disruption of professionals’ beliefs due to prolonged exposure to traumatic events that challenge their worldview (Janoff-Bulmann, 1992). Individuals typically hold positive beliefs about the world and themselves, but when these assumptions are undermined, it can lead to a sense of derailment (Joseph, 2018). In the context of ICU, the dissonance between the emotions felt due to exposure to patients’ suffering and the emotions that they need to express to deal with other tasks (i.e., communicating with physicians) may contribute to this derailment. Emotional dissonance is intimately related to cognitive dissonance regarding identity, which could facilitate these shattered assumptions in the ICU context. Moreover, studies have revealed that emotional dissonance activates a regulation pattern that can lead to surface acting, which involves suppressing genuine emotions and exerting emotional effort (Sommerfeldt and Kent, 2020). Thus, we hypothesized that the demand for emotional effort in ICU could be positively related to shattered assumptions.

Finally, the symptomatology dimension is as associated with symptoms related to Posttraumatic-Stress Disorder, including intrusion, avoidance, and arousal/anxiety in response to feared stimuli (Lee et al., 2018). This dimension includes behaviors (e.g., avoiding trauma-related tasks), cognitions (e.g., ruminating about delivering bad news to relatives), and emotions (e.g., anxiety related to memories of deceased patients) directly related to exposure to specific traumatic stressors (Moreno-Jiménez et al., 2020). Previous studies suggest that symptomatology appears in the short-term due to the direct impact of stressors without the use of personal resources to mitigate the effects. This constitutes the core principle of the Job Demands-Resource Theory (Bakker et al., 2004), where a general loss of energy occurs when demands exceed available resources. However, if exposure to stressors continues, it could lead to specific trauma symptomatology following the CoR theory (Hobfoll, 2001, 2011). We hypothesized that this dimension represents a short-term symptom of energy loss in overcoming emotional effort and the need to maintain a high-quality job performance (Amarnani et al., 2019).

### The present study

Considering the above, we aim to investigate whether emotional effort experienced in an ICU context constitutes an emotional demand that could lead to the three dimensions of STS and diminish vitality. We propose a diary methodology as a novel approach to test the effect of working hours on the well-being of nursing professionals, testing spillover effects to examine the impact of work on how they feel at the end of the day (Ohly et al., 2010). Moreover, most diary studies have focused on establishing predictive models of daily variables and outcomes (Xanthopoulou et al., 2012; Bakker and Albrecht, 2018; Benson and Bruner, 2018; Cambier et al., 2019), but few have focused on the fluctuations of these demands and their predictive value for outcomes. Broadly speaking, employing latent profile analysis using longitudinal data allows us to identify unobserved groupings that capture temporal trends throughout the week (Brondino et al., 2020). Through this diary methodology, we seek to study different profiles of emotional effort to observe the fluctuations in this emotional demand and its impact on nurses’ well-being. The daily information provides an in-depth exploration into the phenomenological process that occurs during a week in the working life of a nurse. Furthermore, we aim to test whether these different profiles predict the different dimensions of STS and vitality.

Thus, we formulated the following hypotheses:

*H1*: Different profiles of the level of daily emotional effort will be identified over five consecutive days.

*H2*: High-level emotional effort profiles predict higher levels of daily (a) compassion fatigue, (b) shattered assumptions, and (c) symptomatology compared with low-level emotional effort profiles.

*H3*: High-level emotional effort profiles predict lower levels of daily vitality compared to low-level emotional effort profiles.

## Materials and methods

### Design and procedure

This study employed a diary approach using repeated measures for five consecutive days twice per day. The sample was recruited using the snowball technique by contacting the main supervisors of the ICU in three different hospitals. Once we established contact with the hospital, a clinical session was provided to explain the main goal of the study. They were then given a package that included: (a) a letter describing the goal of the study and confirming anonymity and confidentiality; (b) instructions regarding questionnaire completion; and (c) the daily questionnaires. They were asked to complete the questionnaire twice daily for five consecutive working days. The assessments were as follows: (1) at work, before leaving the workplace, and (2) at home, when time had passed after work, either before going to sleep for nurses on morning shifts or the next morning for those on afternoon shifts. This procedure allowed us to control for the shift variable and collect data, leaving enough space between working and recovery times to examine the impact of working hours when nurses are involved in other activities. Moreover, this time lapse between the predictor and outcomes prevented us from committing response tendencies and common method biases, as suggested by other studies (Podsakoff et al., 2003). As this study consisted of a diary study, including a total of 44 nurses over five days, we counted a total of 220 observations. The University Ethics Committee approved the protocol and assessment (reference number: CEI 71–1,276). This study has not been registered.

### Measures

The measurements were performed twice a day for five consecutive working days. First, we assessed the following emotional job demands:

#### Daily emotional effort at work

This variable was assessed using the Emotional Effort Questionnaire (Quiñones-García et al., 2013). This scale evaluates the extent to which professionals find it challenging to comply with workplace rules governing emotional expression, thereby hindering their job performance (e.g., “How often have you felt that meeting the rules of emotional expression directly impacted on your work in other tasks?”). To assess it on a daily basis, the items were rewritten, incorporating the phrase “today at work” at the beginning. It consisted of a 7-item scale with responses ranging from 1 “never” to 5 “always.” The reliability index, measured through Cronbach’s Alpha, averaged 0.86, ranging from 0.84 to 0.87.

Second, we assessed the following outcomes at home:

#### Daily compassion fatigue

This variable was assessed with the Secondary Traumatic Stress Scale (STSS) using the Spanish version validated by Meda et al. (2011). To assess it on a daily basis, the items were rewritten, with the word “today” added at the beginning. This scale consists of five items that assess the degree of psychological and mental exhaustion of professionals (e.g., “I feel emotionally without strength”). The 5-item scale response ranged from 1 (“totally disagree”) to 4 (“totally agree”). The reliability index based on Cronbach’s Alpha was good, with an average of 0.82, ranging from.73 to 0.87.

#### Daily shattered assumptions

This variable was also assessed with the STSS (Meda et al., 2011) using a 4-item scale to specifically evaluate the changes in beliefs that occur due to exposure to traumatic stimuli (e.g., “my work makes me see the world as unfair”). To assess it on a daily basis, the items were rewritten, with the word “today” added at the beginning. The scale response was the same as mentioned above, ranging from 1 (“totally disagree”) to 4 (“totally agree”). We found a good reliability index using Cronbach’s Alpha, with an average of 0.68, ranging from 0.60 to 0.76.

#### Daily symptomatology

This variable was obtained from the last five items of the STSS scale (Meda et al., 2011). It assesses the degree of symptomatology and individual/social consequences that professionals may suffer due to prolonged exposure to traumatic stimuli (e.g., “I even remember the name of some patients”). To assess it on a daily basis, the items were rewritten, with the word “today” added at the beginning. Responses ranged from 1 (“totally disagree”) to 4 (“totally agree”). We found a good reliability index using Cronbach’s Alpha, with an average of 0.85, ranging from 0.79 and 0.88.

#### Daily subjective vitality

This variable was assessed using the Spanish version of Ryan and Frederick’s Vitality Scale (Ryan and Frederick, 1997; Rodríguez-Carvajal et al., 2010). It consists of seven items that establish the degree to which professionals feel vigorous and alive in different domains (e.g., “I feel such full of energy that seems I am going to explode”). To assess it on a daily basis, the items were rewritten, with the word “today” added at the beginning. A 7-item scale was used to responses, ranging from 1 (“not at all”) to 7 (“very much”). Cronbach’s Alpha was employed to assess reliability, with an average of 0.82, ranging from 0.77 to 0.89.

### Participants

As mentioned above, the sample comprised 44 ICU nurses, with 11 males and 33 females. Concerning shifts, 18 were from the morning shift, 14 from the afternoon shift, and 12 from both shifts. The average age was 39.41 years old. The diary study allowed us to capture a total number of 220 daily observations (44 participants × 5 days).

### Statistical analysis

First, SPSS was used to obtain descriptive statistics. Subsequently, latent profiles of emotional effort were explored using R Studio and the tidy Latent Profile Analysis (LPA) package, considering the levels of emotional effort recorded over five consecutive days.

LPA is a statistical technique used to identify unobserved subgroups or latent profiles within a population, based on observed variables. Tidy LPA maximizes the log-likelihood function to estimate the parameters of a latent profile model. To achieve this, the package employs an iterative optimization algorithm that seeks to determine the parameter values that yield the highest log-likelihood. By maximizing the log-likelihood, the tidy LPA effectively identifies the optimal configuration of the latent profiles for the given dataset. To avoid local maxima, which can lead to suboptimal results, tidy LPA utilizes multiple random starts during the optimization process. This implies that the algorithm starts the optimization from various initial parameter values, allowing it to explore different regions of the parameter space. By considering multiple starting points, a tidy LPA increases the likelihood of finding the global maximum of the log-likelihood function, thereby reducing the risk of being trapped in the local maxima. By employing this procedure, tidy LPA enhances the robustness and accuracy of the latent profile analysis, ensuring that the estimated profiles are more likely to represent the true underlying structure of the data.

To determine the number of profiles, we considered different indicators (Nylund et al., 2007; Tein et al., 2013), including the Bayesian Information Criterion (BIC), Akaike Information Criterion (AIC), and entropy indicator. BIC and AIC should provide low values, whereas entropy should provide values higher than 0.80. We employed a Bootstrap Likelihood Ratio Test (BLRT) to compare the different models.

Finally, to examine whether these latent profiles are associated with outcomes (i.e., daily compassion fatigue, shattered assumptions, symptomatology, and vitality), we employed a hierarchical modeling approach (HLM). Our data presented two levels: level 1 was day-level (*N* = 220 occasions) and level 2 was a person-level measure (*N* = 44 participants) (Nezlek, 2007). The latent profile variable was added at the person-level centered on the grand mean as established by Ohly et al. (2010). In our model, the outcome variables (i.e., emotional exhaustion and vitality) served as day-level variables, while gender, shift, age, and latent profile were considered person-level variables. The R package was used to perform this multilevel analysis.

## Results

### Descriptive analysis

Descriptive analyses are shown in Table 1. As observed, emotional effort had a medium-high score (*M* = 2.88), as well as the three dimensions of STS, with the highest in symptomatology, followed by shattered assumptions. Vitality had a medium-low score (*M* = 3.30). First, we obtained a positive and significant correlation between the three dimensions of STS (i.e., compassion fatigue, shattered assumptions, and symptomatology) and emotional effort. Conversely, this correlation was negative in the case of vitality.

**Table 1:** Means, standard deviations, and correlations of the study variables.

	*M*	SD	ICC	1	2	3	4	5	8
1. Daily emotional effort	2.88	0.79	0.69	1					
2. Daily compassion fatigue	1.91	0.69	0.47	0.25**	1				
3. Daily shattered assumptions	2.28	0.67	0.62	0.40**	0.44**	1			
4. Daily symptomathology	2.70	0.67	0.68	0.41**	0.13	0.44**	1		
5. Daily vitality	3.30	1.24	0.61	−0.15*	−0.28**	0.27**	0.07	1	

ICC, Intra Class Correlation; *p < 0.05; **p < 0.01.

### Hypothesis testing

Second, to explore the latent profiles of emotional effort, as established in H_1_, we examined the statistics for different profile solutions (Table 2). The three-profile solution emerged as the optimal choice, with the lowest BIC and AIC, and the highest entropy.

**Table 2:** Latent profile analysis of emotional effort: criteria values for different profile solutions.

Number of profiles	AIC	BIC	Entropy	BLRT (*p-value*)
2	9386.47	9440.77	0.81	*p* < 0.05
**3**	**9252.40**	**9327.06**	**0.85**	***p* < 0.05**
4	9418.50	9513.52	0.83	*p* < 0.05

AIC, Aikaike Information Criterion; BIC, Bayesian Information Criterion; BLRT, Bootstrapped Likelihood Ratio Test. Bold values = better profile solution.

**Figure 1:**
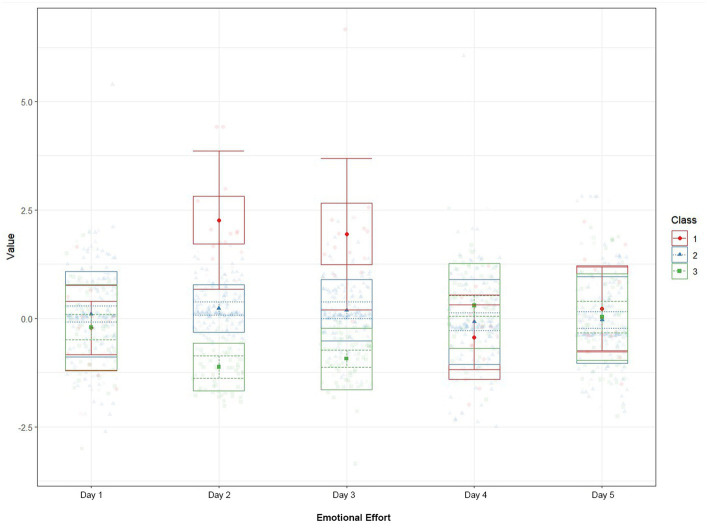
Latent profile analysis of emotional effort: plot of the three-profile solution.

Upon selecting the best profile solution, a plot was obtained to visualize the profiles (Figure 1). The plot illustrates a three-profile solution representing distinct patterns of emotional effort within the study population. Each profile was characterized by different levels of emotional effort, with Profile 1 showing high emotional effort, Profile 2 exhibiting moderate emotional effort, and Profile 3 displaying low emotional effort.

#### Latent profiles predicting secondary traumatic stress and vitality

Finally, to check whether these latent profiles are predictors of daily secondary traumatic stress (H_2_) and daily vitality (H_3_), we ran a hierarchical linear regression with R to maintain the daily level of the measures (within-person) nested in individuals (between-person level). Table 3 presents the results of these analyses. The results obtained were as follows:

**Table 3:** Multilevel estimates for predicting daily outcomes after work (*N* = 44* 5 days = 220 statistical observations).

Variables	Emotional effort^1^			Compassion fatigue^2^			Shattered Assumptions^3^			Symptomathology^4^			Vitality^5^		
	Estimate	SE	*t*	Estimate	SE	*t*	Estimate	SE	*t*	Estimate	SE	*t*	Estimate	SE	*t*
(Intercept)	3.54	0.64	5.49***	1.76	0.48	4.77***	0.92	0.52	1.79	1.24	0.40	3.11**	1.87	0.58	3.19**
Gender	−0.62	0.20	−3.12**	−0.39	0.18	−4.06***	−0.19	0.16	−1.91	0.11	0.19	0.62	0.07	0.27	0.25
Shift	0.12	0.10	1.71	−0.33	0.15	−2.41*	−0.17	0.08	−2.11*	−0.27	0.09	−2.81*	0.40	0.14	2.89**
Age	0.01	0.01	0.13	0.01	0.01	0.95	0.02	0.01	3.48***	0.02	0.01	2.32*	0.02	0.01	1.33
Latent Profiles	0.12	0.13	0.98	0.23	0.19	0.13	0.39	0.11	3.43***	0.38	0.12	3.12**	0.24	0.21	1.198
R^2^	0.03			0.12***			0.14***			0.10***			0.14***		

^1^Null model: Estimate = 2.83; SE = 0.11; *t* = 26.43***; Level 1 Intercept variance (SE) = 0.42; Level 2 Intercept variance (SE) = 0.19. ^2^Null model: Estimate = 1.87; SE = 0.083; *t* = 22.49***; Level 1 Intercept variance (SE) = 0.23; Level 2 Intercept variance (SE) = 0.25. ^3^Null model: Estimate = 2.26; SE = 0.089; *t* = 25.34 ***; Level 1 Intercept variance (SE) = 0.29; Level 2 Intercept variance (SE) = 0.17. ^4^Null model: Estimate = 2.67; SE = 0.10; *t* = 25.58***; Level 1 Intercept variance (SE) = 0.39; Level 2 Intercept variance (SE) = 0.18. ^5^Null model: Estimate = 3.31; SE = 0.15; *t* = 22.02***; Level 1 Intercept variance (SE) = 0.81; Level 2 Intercept variance (SE) = 0.51. *p < 0.05; **p < 0.01; ***p < 0.001.

##### Daily compassion fatigue

To test H_2a_, we obtained that gender and shift were negative significant predictors of daily compassion fatigue (*E* = −0.396, *SE* = 0.178; *t* = −2.214; *p* < 0.05; and *E* = −0.33, *SE* = 0.155, *t* = −2.131, *p* < 0.05, respectively). None of the latent profiles of emotional effort was a significant predictor. The adjusted R-squared value explained 12% of the between-person variance through this predictive model. Additionally, the intercept was 1.76, meaning that this was the average score for daily compassion fatigue with lower levels of demographic variables (men, morning shift, Profile 1 in emotional effort, and average age).

##### Daily shattered assumptions

Concerning H_2b_, it seems that shift and age were significant predictors of daily shattered assumptions (*E* = −0.123, *SE* = 0.054; *t* = −2.254; *p* < 0.05; and *E* = 0.018, *SE* = 0.005, *t* = 3.733, *p* < 0.001, respectively). Moreover, we found the latent profiles to be significant and positive (*E* = 0.492, *SE* = 0.115, *t* = 4.271, *p* < 0.001). The adjusted R-squared value revealed that this model could predict 14% of the total explained between-person variance. Moreover, the intercept was 0.92, meaning that this was the average punctuation in daily shattered assumptions with lower levels of demographic variables (men, morning shift, Profile 1 in emotional effort, and average age).

##### Daily symptomatology

Concerning H_2c_, we found gender and shift to be significant predictors (*E* = 0.571, *SE* = 0.115, *t* = 2.345, *p* < 0.05; *E* = −0.155, *SE* = 0.064, *t* = −2.428, *p* < 0.05, respectively). Moreover, latent profiles appeared to be positive and significant predictor (*E* = 0.436, *SE* = 0.136, *t* = 3.193, *p* < 0.01). This model allowed us to explain 10% of the between-person variance. Additionally, the intercept was 1.24, meaning that this was the average punctuation in daily symptomatology when lower levels of demographic variables were established, as previously stated.

##### Daily vitality

Finally, regarding H_3_, the only predictors that resulted in being significant were shift (*E* = 0.352, *SE* = 0.093, *t* = 3.777, *p* < 0.001) and age (*E* = 0.021, *SE* = 0.008, *t* = 2.559, *p* < 0.05). This model explained a total of 14% between-person variance. Concerning the intercept, the value was 1.87, indicating that this was the average punctuation in daily vitality when the following categories were met: men, morning shift, Profile 1 in emotional effort, and average age.

Additionally, the intercept values were significant in all adjusted models except for the model explaining daily shattered assumptions. The intercepts represent the baseline level of the dependent variable when the predictors included in the model have a value of zero. This means that the expected daily levels of emotional effort (*E* = 3.30, *p* < 0.01), compassion fatigue (*E* = 1.70, *p* < 0.001), symptomatology (*E* = 1.24, *p* < 0.01) and vitality (*E* = 1.87, *p* < 0.01) were significantly different from zero, even in the absence of predictor effects.

## Discussion

This study highlights current worldwide phenomena such as psychosocial risks and STS experienced by nursing professionals, especially after the COVID-19 pandemic outbreak. Martin-Rodriguez et al. (2022) argued that almost 40% of nursing professionals present severe or moderate symptoms of this psychosocial risk; thus, special attention should be paid to this problem. Moreover, this study is a step forward in daily studies conducted to date. In this sense, the establishment of a three-profile solution provides valuable information on the daily functioning of emotional patterns among nursing professionals in ICU. In addition, the information provided by the three-profile solution allowed us to speculate about the differences in emotional regulation strategies that could occur during working hours in such emotionally demanding contexts. This provides a necessary background for developing future preventative programs. In addition, this study fills a gap in the effect of daily emotional effort in an emotionally demanding work settings (i.e., ICU) and its relationship with STS and vitality, which has been poorly explored in previous studies. Therefore, we obtained interesting results for both outcomes.

First, daily compassion fatigue was predicted only by sociodemographic variables, with no predictive relationship between emotional effort profiles and this outcome. Despite this, the descriptive analysis revealed a positive and significant relationship between compassion fatigue and emotional effort, but a lack of predictive value regarding emotional effort profiles.

However, we found gender and shift were significant negative predictors of daily compassion fatigue, meaning that compassion fatigue seems to be lower in women than in men, especially in the afternoon shift. These results align with previous studies (Moreno-Jiménez et al., 2020), where gender roles were identified as explaining how men may be less accustomed to managing and dealing with emotions compared to women, posing a risk factor of increased daily compassion fatigue levels (Eagly and Wood, 2016). This lack of expertise in managing emotional events may result in a rapid loss of resources in attempting to cope with these emotions, leading to an increase in compassion fatigue. We suggest two possible explanations for this shift. Morning shifts mark the beginning of relatives’ visits and the information provided by physicians. This point suggests that intense and emotionally charged situations may occur during this time, in contrast to the afternoon shift, with possibly fewer relative visits. However, this result could be a consequence of the assessment time, as afternoon shift nurses assessed their level of STS and vitality after a period of recovery the following morning. This recovery time could lead to a decrease in compassion fatigue levels, as supported by previous studies (Sonnentag et al., 2008). We strongly support the latter explanation, as we obtained the same results in shattered assumptions and symptomatology, with the opposite trend noted for vitality.

Notably, we did not find age to be a significant positive predictor, suggesting that this outcome could possibly be more related to the years spent in ICU rather than age itself. This result is supported by previous studies on healthcare professionals in the ICU, where compassion fatigue is not considered a short-term outcome but a medium-and long-term outcome associated with the time spent in the ICU dealing with specific traumatic tasks and the regulation strategies used (Moreno-Jiménez et al., 2020). This observation allows us to differentiate between compassion fatigue and emotional exhaustion, which often overlap in several studies (Cieslak et al., 2014). In contrast, while emotional exhaustion may appear in the short-term as a result of losing energy to overcome challenges (Shoji et al., 2015), compassion fatigue involves specific resources (i.e., empathy) in certain professionals (i.e., helping professionals), as established by Figley (2002).

Second, the daily measurement of shattered assumptions yielded similar results for shift and age. It appears that older nursing professionals are more susceptible to experiencing these disruptions in their beliefs, possibly due to changes in values across their lifespan, where the significance of work becomes particularly relevant (Harris and Tao, 2022). Probably younger nurses place a great deal of value on learning and challenging experiences, while they are building the meaning of work and their life, which is a protective factor for burnout and STS (Harris and Tao, 2022). In contrast, older nurses could have a greater impact on their belief in personal invulnerability (Janoff-Bulmann, 1992; Rodríguez-Muñoz et al., 2010). It is likely that older nurses may experience more negative consequences, and consequently, stressors may have a stronger impact compared to their younger counterparts. Consequently, beliefs of invulnerability and self-worth could easily be threatened, increasing the likelihood of being shattered.

Moreover, an interesting result emerged from the three-profile solution. Specifically, emotional effort profiles are significant positive predictors, suggesting that the greater the daily emotional effort, the less shattered the assumptions. These results could be explained by the type of emotion/cognitive strategies used to self-regulate emotionally demanding situations. In other words, nursing professionals dealing with greater emotional dissonance between what they feel and what they must express may be less focused on cognitive expectations and unity of their identity. Instead, they seem to prioritize regulating these emotions, following the CoR principles, as a loss of emotional resources occurs during the attempt to balance them (Hobfoll, 2001). Previous studies have identified passion for work as a stable resource to maintain a high-quality job in demanding professions (van Mol et al., 2018; Amarnani et al., 2019), particularly in ICU contexts with high demands, leading to lower levels of STS (Moreno-Jiménez et al., 2022). Building on this, nurses with lower emotional effort (Profile 3) may lack the trigger to apply these regulation strategies (i.e., harmonious passion, recovery activities) and could be more vulnerable to experiencing shattered beliefs. In contrast, nursing professionals with high emotional effort (Profile 1) may activate these strategies, allowing them to protect themselves from derailment (Joseph, 2018) and cognitive dissonance (Sommerfeldt and Kent, 2020). Speculating about the use of the strategies activated in Figure 1, we observed peaks in the levels of emotional effort on days two and three, with nursing professionals in Profile 1 showing the highest levels and those in Profile 3 with the lowest. We observed stabilization over the next two days as a result of the strategies applied. Thus, we hypothesized that on days when nursing professionals experience more emotional effort, they may also display lower levels of shattered assumptions, contrary to established in H_2b_.

Third, concerning daily symptomatology, we did not find gender to be a significant predictor, contrary to the results for compassion fatigue. Age could have a greater impact on daily levels of symptomatology than expertise in dealing with emotionally demanding situations and gender differences (Eagly and Wood, 2016). Hence, as occurs in shattered assumptions, older nurses are likely to suffer from symptomatology, possibly linked to this personal vulnerability, not only as a belief but also having physical consequences. Additionally, it is likely that older nurses have more years of experience in the ICU, which means more exposure to stressors and, in turn, higher levels of symptomatology (Moreno-Jiménez et al., 2020). Moreover, as previously mentioned, shift was a significant and negative predictor, possibly due to the timing at which the outcomes were assessed.

Concerning symptomatology, the three profiles were significant and positive predictors, meaning that the higher the profile, the higher the daily symptomatology. In other words, nursing professionals with less emotional effort seemed to possess more daily symptomatology, opposed as stablished in H_2c._ This led us to speculate on the symptomatology dimension, which has been proposed in previous studies as a short-term dimension that appears with prolonged exposure to ICU demands (Moreno-Jiménez et al., 2019, 2020) similar to emotional exhaustion (Bakker and Demerouti, 2017). These findings allow us to hypothesize that even low levels of emotional effort could lead to a high level of symptomatology, possibly related to other variables, such as stressors or rumination about the emotional situations they have to deal with Donahue et al. (2012). As mentioned above, a low emotional effort profile may not trigger the activation of emotion regulation strategies (i.e., recovery activities when facing emotional demands), leading to greater levels of symptomatology. Nursing professionals in Profile 1 seemed to adapt and properly regulate their daily symptomatology, possibly meaning that other factors in high-emotional effort profiles mitigate this as a consequence of being obliged to protect themselves.

Finally, the vitality dimension was not significantly related to any of the three emotional effort profiles, thus we reject our H_3_. Emotional exhaustion seems to be strongly linked to an increase in job demands (Bakker and Demerouti, 2017; Bakker et al., 2023) but vitality may be more closely related to personal resources, such as passion for work, rather than job demands. A study by Mudło (2023) supports this statement, as only medical students without passion presented low levels of vitality, whereas students with high levels of passion presented high levels of subjective vitality. Additionally, a study by Carmona-Cobo and Lopez-Zafra (2022) revealed that daily subjective vitality was predicted by daily social support in a sample of nurses. Thus, these findings highlight the strong relationship between vitality and resources rather than demands.

This study had some limitations that should be addressed in future research. First, the assessment of nurses from different shifts could directly impact the results obtained in the three dimensions of STS, as we tried to equalize this assessment using the same criteria (i.e., assessing emotional effort during working time, and STS and vitality after leaving work). This could be responsible for the lower levels of STS and higher vitality in the afternoon shift, as it was assessed the next morning. In further research, it is advisable to increase the sample size and conduct analyses splitting by shifts to clarify these data. Additionally, in order to avoid potential fatigue and professionals’ tiredness, some of the scale used had few items, which could attempt against its reliability. However, we highlighted that the use of a daily approach with five days of assessment at two moments per day allows us to control the response tendencies of participants and have more observations that enable a more comprehensive understanding of the phenomenon studied. Finally, it is important to remark that this study counts on a small sample size due to the difficulties of assessing this specific sample (only nurses) with a wide assessment protocol (five consecutive days) within a specific work setting (intensive care units). It seems undeniably relevant to replicate this study with bigger sample to gain generalization, and to boost the scientific value providing steps forward the preventative models.

### Theoretical and practical contributions

Overall, these findings emphasize the theoretical contributions that should lead to practical implications, being highly relevant in ICU contexts, especially regarding the mental health of nursing professionals. As emotional demands such as emotional effort may play a significant role in predicting daily levels of shattered assumptions and symptomatology, more resources should be provided to these professionals to deal with this demand. More job resources, such as coworker and leader support, may diminish this emotional effort and increase daily levels of vitality. This fact may allow the nursing professionals to convert their working hours in a positive experience through the role of vitality. Additionally, the findings revealed that those with high-emotional effort profiles are able to self-regulate and overcome this, but those with low-emotional effort profiles present high levels of STS, making it important to teach strategies to deal with this. These results remark the importance of providing cognitive and emotional strategies to manage with such emotional demands, even from the lowest levels. In this sense, implementing job crafting techniques, as supported by Demerouti et al. (2019), could be useful in teaching how to identify emotional demands (i.e., bad news communication and communication with relatives), identify personal resources (i.e., social skills and the establishment of a time-out after communicating), and how to apply these personal resources specifically for each demand. For example, to have the ability of applying assertiveness to effectively communicate to the patient’s relatives and stablishing a recovery time after the difficult communication, as a time-out to breath, and either use a relaxion technique or favor a small psychological detachment. Job crafting could be a valuable way to provide self-control in self-regulation skills, especially when demand levels are lower (Bakker et al., 2023).

## Conclusion

This study emphasized the impact of daily emotional effort as an emotional demand among nurses in ICU, specifically affecting their beliefs and symptomatology related to trauma. Moreover, this study allowed us to delve into the daily fluctuation of emotional demands, providing information about how they could change over a week and the existence of strategies to regulate them. The lack of regulation strategies to overcome this emotional effort at work could lead to a greater level of negative consequences on a daily basis (i.e., shattered assumptions and symptomatology), even after work, which could increase individuals’ intentions to leave the profession. Additionally, job resources should be considered, including social support, which is related to subjective vitality in prior literature. Vitality is an elemental phenomenon that increases nurses’ well-being.

## Data availability statement

The raw data supporting the conclusions of this article will be made available by the authors, without undue reservation.

## Ethics statement

The studies involving humans were approved by Ethical Committee of Autonomous University of Madrid (reference number CEI 71-1276). The studies were conducted in accordance with the local legislation and institutional requirements. The participants provided their written informed consent to participate in this study.

## Author contributions

JM-J: Conceptualization, Data curation, Formal analysis, Funding acquisition, Investigation, Methodology, Project administration, Resources, Software, Validation, Writing – original draft, Writing – review & editing. MR: Conceptualization, Data curation, Formal analysis, Software, Writing – original draft, Writing – review & editing. LB-D: Conceptualization, Funding acquisition, Investigation, Methodology, Resources, Supervision, Validation, Writing – original draft, Writing – review & editing. MH-H: Formal analysis, Methodology, Software, Writing – original draft, Writing – review & editing. EG: Conceptualization, Funding acquisition, Investigation, Project administration, Resources, Supervision, Validation, Writing – review & editing.
